# Lato sensu postgraduate courses in Brazilian medical education: a qualitative study

**DOI:** 10.1590/1806-9282.20251299

**Published:** 2025-12-08

**Authors:** Juliana Sousa, José Eduardo Lutaif Dolci, Mário César Scheffer

**Affiliations:** 1 Universidade de São Paulo, Faculty of Medicine, Department of Preventive Medicine – São Paulo (SP), Brazil.; 2 Brazilian Medical Association, Faculty of Medical Sciences of the Santa Casa of São Paulo, Department of Scientific – São Paulo, (SP), Brazil.; 3 Universidade de São Paulo, Faculty of Medicine, Department of Preventive Medicine, Brazilian Medical Demographics Research Group – São Paulo (SP), Brazil.

**Keywords:** Specialization, Distance education, Physicians, Medical education, Private sector

## Abstract

**OBJECTIVE:**

The aim of this study was to analyze the provision of lato sensu postgraduate courses within the context of specialist medical training in Brazil.

**METHODS:**

Using a qualitative research approach, in-depth interviews were conducted based on a semi-structured guide with 24 key informants occupying strategic roles in the training of medical specialists. Participants included health system managers, medical educators, leaders of professional Medical Societies, and representatives from both public and private sectors.

**RESULTS:**

Four core themes emerged from the interviews regarding lato sensu postgraduate courses in medicine, reflecting key concerns and perceptions raised by participants: the lack of regulation and oversight amid an expanding market; risks to the quality of specialist medical training; the predominance of private-sector provision, often misaligned with the needs of Brazil's Unified Health System; and the importance of preserving the existing framework for specialist certification, as overseen by the National Commission for Medical Residency and the Brazilian Medical Association.

**CONCLUSION:**

Lato sensu postgraduate courses may serve a complementary role in the continuing education of doctors, but they are not a substitute for Medical Residency or the Specialist title granted by the Brazilian Association or Society of the respective specialty, which is affiliated to the Brazilian Medical Association. These programs should be subject to formal regulation and oversight, whether implemented by governmental authorities or delegated to competent civil society organizations.

## INTRODUCTION

In Brazil, current legislation recognizes only two official pathways for physicians to obtain specialist status: Certificate of Completion of Medical Residency accredited by the National Commission for Medical Residency (CNRM) or Specialist title granted by the Brazilian Association or Society of the respective specialty^1^. However, the expanding presence of lato sensu postgraduate (LSPG) courses introduces a set of complexities.

LSPG courses, which are not equivalent to Medical Residency, are defined in national legislation as specialization courses aimed at continuing education, with the purpose of supplementing academic training^2^. The National Council of Education (CNE), whose 2018 regulation about LSPG courses explicitly excludes Medical Residency from its scope—on the grounds that Medical Residency constitutes a postgraduate modality specifically tailored to physicians^3^, has established that LSPG courses are generally open to candidates holding an undergraduate degree, must be registered in the official e-MEC System of institutions and courses, and are required to include a pedagogical plan and a minimum workload of 360 h. Despite this, their provision remains unregulated: they do not require prior authorization and are not subject to oversight or quality assessment by any governmental body.

The role of LSPG courses remains a subject of controversy, largely due to the heterogeneity of the courses offered and the lack of conceptual, regulatory, and evaluative clarity surrounding this level of education^4^.

The insufficient availability of Medical Residency positions to keep pace with the rapid expansion of undergraduate medical education^5^ and government initiatives aimed at increasing the provision of specialized care within the Brazilian Unified Health System (SUS)^6^, among other factors, highlights the need for a more in-depth discussion regarding the profile and adequacy of LSPG courses in specialist medical training.

This article seeks to examine the provision of LSPG courses within the framework of Brazil's current model for training and deploying medical specialists.

## METHODS

The qualitative research involved 24 interviews conducted throughout 2024, each lasting an average of 60 min. All interviews were audio recorded and fully transcribed.

Interviewees were strategically positioned professionals and leaders involved in the training, regulation, and provision of medical specialists. Participants included members of regulatory and certifying bodies, such as the CNRM, State Medical Residency Commissions (Cerems), and Medical Societies affiliated to the Brazilian Medical Association (AMB). The sample also included public-sector managers from governmental health and education agencies, private-sector representatives engaged in medical education, and academic researchers specializing in medical training.

For the selection of study participants, a purposive sampling strategy was employed, prioritizing individuals whose professional trajectories and institutional affiliations aligned with the study's objectives and contributed to ensuring representativeness and diversity (Table 1).

**Table 1:** Criteria for the selection of study interview participants.

Institution or sector of origin of affiliation	Selection criteria
National Commission for Medical Residency (CNRM)	Individuals occupying formally designated strategic positions.
State Medical Residency Commissions (Cerems)	Coordinators from states with the highest number of medical resident positions.
Medical Societies affiliated with the Brazilian Medical Association	Representatives from the six societies with the largest number of certified specialists.
Public-sector health and education agencies	Individuals in formal strategic roles within the CNRM Plenary.
Private-sector institutions involved in medical education	Executives from leading private-sector organizations engaged in physician training.
Academic researchers in medical training	Selected based on academic output and recognized expertise in the field.

Source: Elaborated by the authors based on in-depth interviews using a semi-structured script. DMB, 2025.

Developing a semi-structured interview guide—refined during a pilot phase with six participants, who were included in the total of 24 interviewees—the study aimed to capture opinions and perceptions regarding the role, potential, and risks of LSPG courses in the training of medical specialists.

The data collected were analyzed using thematic content analysis, with the aim of identifying, presenting, and discussing key themes based on selected excerpts from the interviewees’ statements^7–9^.

The study was approved by the Research Ethics Committee (CEP/FMUSP) under CAAE Opinion No. 71626323.8.0000.0068, dated August 11, 2023.

## RESULTS

Four main themes (Table 2) emerged from the analysis of the interviews, reflecting core concerns and perspectives regarding LSPG courses in medicine: (1) the lack of regulation and oversight amidst their rapid expansion; (2) risks to the quality of specialist medical training; (3) the predominance of private-sector provision misaligned with the needs of the Unified Health System (SUS); and (4) the importance of preserving the existing framework for specialist certification, coordinated by the CNRM and the AMB.

**Table 2:** Main themes, key issues, and selected quotes from interviewees.

Themes	Key issues	Selected quotes from interviewees
1. LSPG courses: Regulation and oversight	Unclear roles of the federal government and medical associations in the regulation of LSPG courses Litigation to grant specialist status to physicians completing LSPG courses	We need a new regulatory framework for specialisation that ensures universities retain their autonomy and allows individuals to pursue complementary training pathways. (…) There is no problem with professionals undertaking a LSPG specialisation. The issue lies in how we regulate certification (Public sector representative). These programmes are recognised by the Ministry of Education as postgraduate courses, but they cannot be promoted as courses that confer specialist status. Some do advertise them this way, and in such cases, we take legal action (Representative of a medical society). I'm very sorry that these matters end up in Court, but those primarily responsible are the ones who should reflect on whether they should pursue that path in the first place. It's not a legitimate path, it's a shortcut to obtaining the specialist title. Consider the difference in time, structure and opportunity between a hands-on residency programme and one that is purely theoretical, held on weekends, or even delivered remotely (Medical education specialist).
2. LSPG courses: Quality of specialized medical training	Incompatible workload and formats with quality standards Lack of a pedagogical framework and a competency matrix	[About the LSPG course] How many faculty members are there? Publications? Qualifications? Workload? Student-to-teacher ratio? Hospital? Laboratory? Library? This demands a thorough evaluation (Representative of a medical society). We will have to change our approach and stop offering low-quality products instead providing high-quality [LSPG] courses so that they begin to gain a good reputation. Someday, one of our students may prefer to pursue a top-tier postgraduate LSPG course rather than a substandard residency (Private sector representative). There are no simulation models that can replace direct patient experience [as occurs in Medical Residency]. The safety level of a graduate who has completed a 2,880-h residency is markedly different from that of someone who underwent remote training totaling 360 h. Direct comparisons are not feasible (Public sector representative).
3. LSPG courses: Commercialization of specialist medical training	Unregulated commercial provision, predominantly costly and fee-based Limited provision in public institutions and weak alignment with SUS needs	Investing in a physician means investing in 50 potential years of product consumption: getting into medical school, paying for school itself, entering residency, the various LSPG courses they'll pay for later, and continuing medical education (Medical education specialist). [About] Private courses, entrepreneurs see a market niche and are creating training opportunities supported by strong marketing strategies on social media, showcasing "success stories". This has attracted an audience in Brazil that is actively seeking such options, especially since not everyone is entering residency (Representative of a medical society). We have the SUS, what's missing now is the work to build on that. (…) I would like to see any educational group truly adopt the Medical Residency model and succeed in training specialists through that approach. It doesn't matter whether it's public or private, as long as it follows the scientific and ethical principles of the health professions. Will LSPG courses also begin to prioritise the fields most needed to better meet social needs? (Public sector representative)
4. Model for specialist certification and credentialing	Medical Residency as the gold standard in specialist training Medical Societies as certifying bodies for specialist titles LSPG courses as a tool for the continuing medical education of doctors	Specialist training through residency programs constitutes the gold standard. This model has stood the test of time, remaining exemplary in the education of medical specialists by immersing trainees in clinical practice, fostering opportunities for peer discussion, ensuring regular supervision, and exposing them to professional role models. Undoubtedly, there is a need for a comprehensive review of the entire system. However, the overarching aim should be to broaden access to such training, rather than maintain the current level of restriction (Medical education specialist). With regard to the ability of these professionals to obtain specialist title, it is noteworthy that this group [trained through LSPG] is consistently among those with the highest failure rates in the board examinations administered [by Medical Societies] (Representative of a medical society). There is a clear role for LSPG in the context of continuing professional development, particularly for doctors, who have completed their formal training, hold specialist title and seek to update their skills in response to emerging technologies or practices, such as the introduction of robotic-assisted surgery (Private sector representative).

Source: Elaborated by the authors based on in-depth interviews using a semi-structured script. DMB, 2025. LSPG: lato sensu postgraduate.

## DISCUSSION OF THE RESULTS

The interviews highlighted the need to strengthen the regulatory and oversight mechanisms governing LSPG courses. The proliferation of these programs—often regarded as controversial—calls for the establishment of a formal regulatory and evaluation framework capable of ensuring quality assurance and accreditation. This is also seen as a necessary step to address the growing number of legal claims seeking to grant physicians holding LSPG certificates the right to advertise themselves as specialists.

In contrast to Medical Residency programs, the absence of curricular guidelines and validated pedagogical frameworks in LSPG courses—along with a lack of rigor in course formats (including the widespread use of distance learning) and inconsistencies in workload and content—were identified as factors undermining the quality of medical training and posing potential risks to patient safety.

LSPG courses are heterogeneous in structure and content, and diverge significantly from the standards established by the CNRM and the AMB, currently the only two legally recognized pathways for specialist training (ENDNOTE). In distance-learning or hybrid formats, the lack of supervised practical training further exacerbates concerns regarding the ability of LSPG courses to ensure the acquisition of essential clinical competencies required for safe and effective professional practice.

Subject to unrestricted commercial supply, the encroachment of market-driven interests into medicine^10–12^, including the specialized training phase, was highlighted by several interviewees. They expressed concerns regarding the lack of quality assurance and the insufficient prioritization of scientific and ethical principles within the educational process. The involvement of education-sector business groups at all stages of medical careers encompasses LSPG courses, preparatory courses for Medical Residency, and other services, driven by commercial objectives that are often misaligned with the needs of the Brazilian Unified Health System (SUS).

The interviewees emphasized that Medical Residency and Specialty titles granted by Medical Societies should be strengthened and refined, as they jointly play an indispensable and irreplaceable role in specialist training, particularly in the current context of increasing medical school enrollment and course offerings that remain insufficient relative to the number of residency positions available. The need was highlighted for an in-depth understanding of the factors driving some medical graduates to seek a "shortcut" through LSPG courses instead of opting for formal training via Medical Residency.

It is incumbent upon public authorities, in collaboration with medical associations, to regulate LSPG certification while preserving the value of specialist titles and aligning its provision with the strategic needs of the Brazilian Unified Health System (SUS). The potential role of LSPG in complementing and updating already-certified specialists was noted, particularly in remaining aligned with scientific advancements and the incorporation of new technologies within medical specialties.

## CONCLUSION

The proliferation of LSPG courses may pose risks to the quality of specialist medical training and should therefore be subject to regulatory oversight, whether governmental or delegated to civil society.

LSPG courses may contribute as a complementary component within the broader framework of continuing medical education, but they should not be considered a substitute for Medical Residency or certification through the specialist title exam by the AMB.

Public policies in health and education must prioritize the quality of specialist training, the safety of the population, and the strategic needs of the Unified Health System (SUS).

## Data Availability

The datasets generated and/or analyzed during the current study are available from the corresponding author upon reasonable request.
